# The Effect of Lumbar Lordosis on Screw Loosening in Dynesys Dynamic Stabilization: Four-Year Follow-Up with Computed Tomography

**DOI:** 10.1155/2015/152435

**Published:** 2015-12-08

**Authors:** Chao-Hung Kuo, Peng-Yuan Chang, Tsung-Hsi Tu, Li-Yu Fay, Hsuan-Kan Chang, Jau-Ching Wu, Wen-Cheng Huang, Henrich Cheng

**Affiliations:** ^1^Department of Neurosurgery, Neurological Institute, Taipei Veterans General Hospital, 17F, No. 201, Shih-Pai Road, Sec. 2, Beitou, Taipei 11217, Taiwan; ^2^School of Medicine, National Yang-Ming University, Taiwan; ^3^Molecular Medicine Program, Taiwan International Graduate Program (TIGP), Academia Sinica, Taipei, Taiwan; ^4^Institute of Pharmacology, National Yang-Ming University, Taiwan

## Abstract

*Introduction*. This study aimed to evaluate the effects of Dynesys dynamic stabilization (DDS) on clinical and radiographic outcomes, including spinal pelvic alignment.* Method*. Consecutive patients who underwent 1- or 2-level DDS for lumbar spondylosis, mild degenerative spondylolisthesis, or degenerative disc disease were included. Clinical outcomes were evaluated by Visual Analogue Scale for back and leg pain, Oswestry Disability Index, and the Japanese Orthopedic Association scores. Radiographic outcomes were assessed by radiographs and computed tomography. Pelvic incidence and lumbar lordosis (LL) were also compared.* Results*. In 206 patients with an average follow-up of 51.1 ± 20.8 months, there were 87 screws (8.2%) in 42 patients (20.4%) that were loose. All clinical outcomes improved at each time point after operation. Patients with loosened screws were 45 years older. Furthermore, there was a higher risk of screw loosening in DDS involving S1, and these patients were more likely to have loosened screws if the LL failed to increase after the operation.* Conclusions*. The DDS screw loosening rate was overall 8.2% per screw and 20.4% per patient at more than 4 years of follow-up. Older patients, S1 involvement, and those patients who failed to gain LL postoperatively were at higher risk of screw loosening.

## 1. Introduction

Sciatica, neurogenic claudication, and lower back pain are common symptoms of degenerative lumbar spondylolisthesis. Although many patients who experience these can be managed with medication or rehabilitation, spondylolisthesis with spinal stenosis at L4/5 is not uncommon in the elderly and sometimes requires surgery [1, 2]. The surgical options usually include decompression and stabilization if there is segmental instability. Moreover, during the past several decades, various surgical corridors have been developed, including anterior [3], posterior [4, 5], and lateral [6], via traditional open, minimally invasive [4, 5, 7], or endoscopic [8] approaches.

In the last decade, there has been an emerging option of spinal motion preservation surgery (SMPS) for lumbar spondylosis. Unlike fusion, preservation of motion of the indexed spinal segments after surgical decompression intuitively allows movements similar to one's physiology motion. As long as adequate spinal stability is achieved, it theoretically provides favorable outcomes and eliminates the development of adjacent segment disease (ASD) after arthrodesis [9]. However, the actual benefits of these SMPS still require further studies to corroborate.

In 1994, Dr. Dubois first used the Dynesys dynamic stabilization (DDS, Zimmer Spine, Minnesota) [10]. The pedicle screw based system was intended to provide mobile stabilization, controlling motion in all three planes (flexion/extension, axial rotation, and lateral bending). Its safety and efficacy have been demonstrated by several case series for the management of degenerative disc disease (DDD), lumbar spondylosis, and spondylolisthesis [10–15]. On the other hand, restoration of sagittal balance, lumbar lordosis (LL), and pelvic incidence (PI) were reportedly correlated with clinical improvement after fusion of the thoracolumbar spine [16, 17]. Nevertheless, the true effect of stabilization for patients with slight to mild disability remains uncertain. Furthermore, there is a paucity of the literature addressing these spinal pelvic parameters, including sagittal balance, LL, and PI, in patients with spondylolisthesis who were managed with dynamic stabilization. Therefore, this study aimed to investigate the clinical outcomes of DDS and its correlation to the radiological parameters in the setting of spondylolisthesis.

## 2. Methods

### 2.1. Patient Population

Consecutive patients who underwent posterior decompression and Dynesys dynamic stabilization (DDS) in the authors' institute from 2006 to 2010 were included. All their medical records, radiological studies, and clinical evaluations were retrospectively reviewed.

The inclusion criteria were symptomatic lumbar spinal stenosis, Meyerding grade one spondylolisthesis, recurrent disc herniation, and degenerative disc disease causing symptoms such as neurogenic claudication, back pain, leg pain, or any combination of the above. All patients failed at least 12 weeks of conservative management, including medication, traction, local injection, or physical therapy. The exclusion criteria were multiple level disc disease, spondylolisthesis of Meyerding grade two or higher, degenerative scoliosis, the presence of vertebral fracture, infection, tumor, or loss of follow-up. Every patient was evaluated by anteroposterior radiograph, lateral dynamic (i.e., flexion and extension) radiographs, computed tomography (CT), and magnetic resonance imaging (MRI) prior to the operation.

### 2.2. Surgical Technique

Patients were placed under general anesthesia in a prone position with adequate cushioning. Standard total laminectomies were performed cautiously with preservation of the facet joints. Subdermal dissection was made through the same midline skin incision, which allowed another two fascial incisions, one on each side, for the Wiltse approach. The Dynesys titanium alloy screws without hydroxyapatite coating were then placed transpedicularly through the Wiltse plane without destruction of the facet joints. The DDS constructs, polycarbonate-urethane spacers, and polyethylene-terephthalate cords (Sulene-PET) were assembled under appropriate tension, measured by the standard instrument, without specific attempt to reduce the spondylolisthesis intraoperatively. Lateral fluoroscopy was routinely used to assure optimal positioning of the screws at the end of surgery.

### 2.3. Clinical and Radiographic Evaluations

All medical records and radiological images were retrospectively reviewed. Functional outcomes were evaluated by Visual Analogue Scale (VAS) for back and leg pain, Oswestry Disability Index (ODI), and clinical symptom scores of the Japanese Orthopaedic Association (JOA) by special nurses under the guidance of attending surgeons during clinical visits according to the designated time schedule. The patients themselves completed a questionnaire preoperatively and regularly at 6, 12, 18, and 24 months postoperatively.

Lateral standing lumbar radiographs were taken for every patient preoperatively and at the last follow-up. Lumbar lordosis (LL) was defined as the angle measured between the superior endplate of L1 and the superior endplate of S1. Pelvic incidence (PI) was defined as the angle subtended by a line drawn between the center of the two femoral heads and the sacral endplate and a line drawn perpendicular to the sacral endplate. Screw position was defined by postoperative computed tomography (CT) examination. Screw loosening was defined as the presence of a “halo zone sign” or “double halo sign” on anteroposterior radiographs during follow-up. In cases of equivocal findings of screw loosening by the radiographs, multidetector CT scans with two-dimensional reformatted images were used to determine questionable screw loosening.

### 2.4. Statistical Analysis

Data are presented as the average ± standard deviation for continuous variables and as frequency and percentages for categorical variables. All statistical tests were two-tailed, and *p* < 0.05 was considered statistically significant by independent *t*-test or chi-square test. All statistical analyses were performed using MedCalc Software (Ostend, Belgium).

## 3. Results

### 3.1. Patients' Demographic Data

From 2007 to 2010, a total of 291 consecutive patients who underwent 1- or 2-level DDS were included in the present study. Among them there were 206 patients (71%), in whom 1064 screws were placed and who completed the clinical and radiological evaluations for more than 2 years postoperatively and were thus analyzed.

Of these 206 patients, there were 115 men (55.8%) and 91 women (44.2%), and the mean age was 61.0 ± 12.9 years at the time of surgery. The mean clinical follow-up duration was 51.1 ± 20.8 months, and the mean radiological follow-up duration was 40.7 ± 19.7 months. Of the 206 patients, 86 (41.7%) underwent 1-level surgery, and 120 (58.3%) underwent 2-level surgery. The distributions of indexed levels are presented in Table 1.

### 3.2. Clinical Outcomes

**Figure 1:**
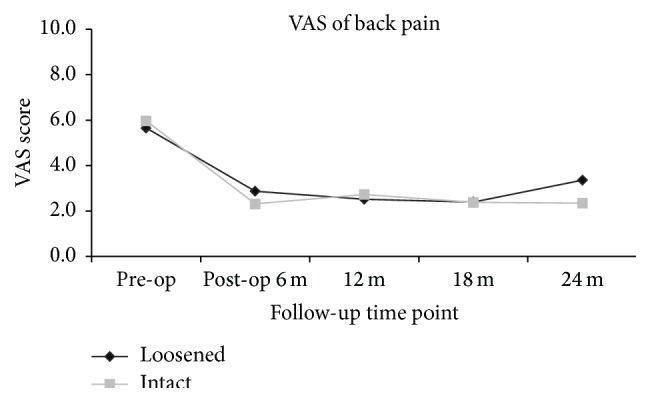
Clinical outcomes measured by VAS for back pain at different time points, suggestive of significant improvement postoperatively. No statistical differences were present between patients with and without screw loosening at each time point.

**Figure 2:**
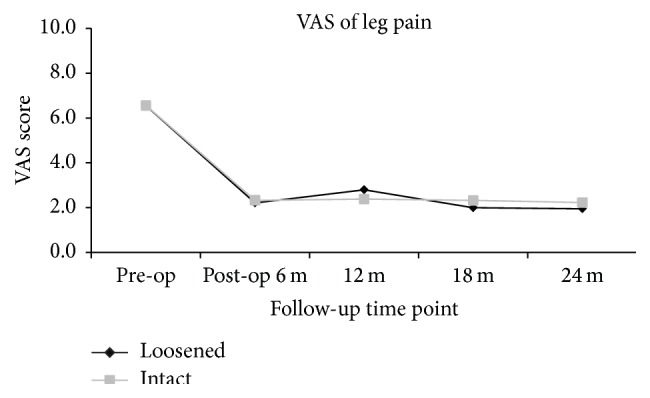
Clinical outcomes measured by VAS for leg pain at different time points, suggestive of significant improvement postoperatively. No statistical differences were present between patients with and without screw loosening at each time point.

**Figure 3:**
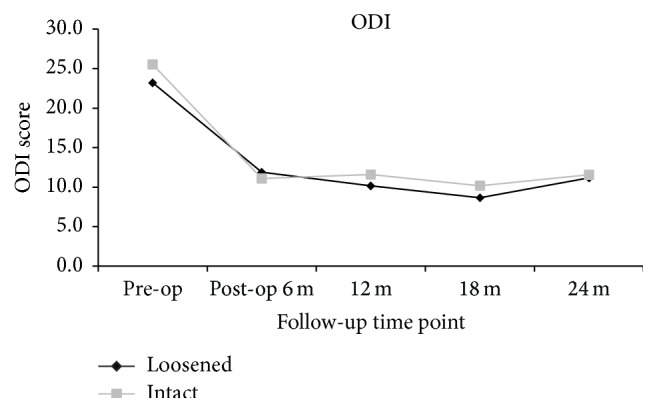
Clinical outcomes demonstrated by ODI at different time points, suggestive of significant improvement postoperatively. No statistical differences were present between patients with and without screw loosening at each time point.

**Figure 4:**
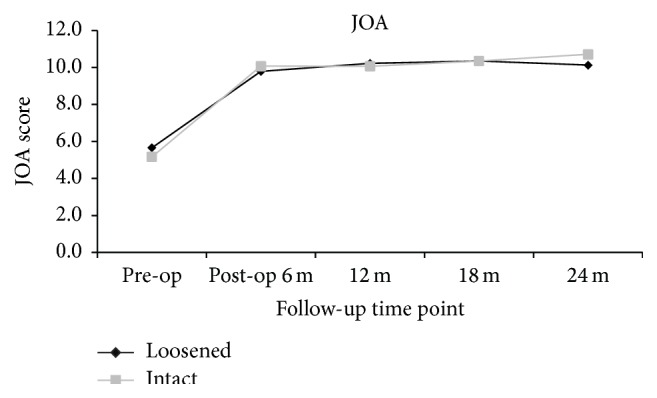
Clinical outcomes demonstrated by JOA score at different time points, suggestive of significant improvement postoperatively. No statistical differences were present between patients with and without screw loosening at each time point.

The clinical outcomes were measured by VAS score of back and leg pain and ODI and JOA scores. When compared to the preoperative status, all the outcome scores had significantly improved at 6, 12, 18, and 24 months after surgery (*p* < 0.05). Whether screw loosening was evident or not, the scores of VAS back and leg pain (Figures 1 and 2), ODI (Figure 3), and JOA (Figure 4) all improved when compared to that of preoperation.

At the 24-month follow-up time point, all clinical scores of patients with screw loosening had significant improvement when compared to that of preoperation (VAS back: 3.4 ± 2.8 versus 5.7 ± 3.3; *p* < 0.01; VAS leg: 2.0 ± 2.9 versus 6.6 ± 3.0; *p* < 0.01; ODI: 11.2 ± 9.6 versus 23.2 ± 9.2; *p* < 0.01; and JOA: 10.1 ± 3.0 versus 5.7 ± 3.4; *p* < 0.01). For patients with intact (i.e., no loosening) screws, all clinical scores at 24 months after operation had significant improvement when compared to that of preoperation (VAS back: 2.3 ± 2.7 versus 6.0 ± 3.1; *p* < 0.01; VAS leg: 2.2 ± 2.9 versus 6.6 ± 2.9; *p* < 0.01; ODI: 11.6 ± 10.3 versus 25.5 ± 9.2; *p* < 0.01; and JOA: 10.7 ± 8.9 versus 5.2 ± 3.0; *p* < 0.01). There were no significant differences between the 2 groups at all evaluation time points (*p* > 0.05) (Figures 1–4).

### 3.3. Screw Loosening and Involvement of S1 Segment

**Figure 5:**
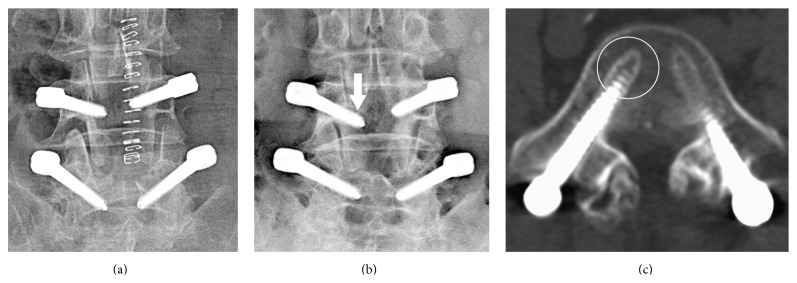
Postoperative images obtained from a 69-year-old female patient who underwent dynamic stabilization at the level of L4/5. (a) Plain radiograph on postoperative day 3. (b) Postoperative plain radiograph 6 months after surgery documented the halo sign (arrow) which was indicative of screw loosening. (c) CT scan performed 19 months after surgery documented the presence of screw loosening (circle).

Among the 1064 screws inserted into 206 patients, radiographic evidence of screw loosening was demonstrated in 87 screws (8.2%) of 42 patients (20.4%) (Figure 5). The mean age of patients with screw loosening was significantly older than that of intact screws (64.6 ± 11.6 versus 60.1 ± 13.1; *p* = 0.03). There were no differences in sex distribution (*p* = 0.99), mean body mass index (*p* = 0.22), diabetes mellitus (*p* = 0.47), hypertension (*p* = 0.47), cigarette smoking (*p* = 0.92), and levels of instrumentation (*p* = 0.48) (Table 2).

Between the two groups of patients (with and without screw loosening), there were no differences in PI, LL, or S1 involvement (all *p* > 0.05). The screw loosening was distributed from L2 to S1, and the highest percentage of screw loosening was found in S1 (16.3%).

Among the 46 patients whose DDS construct involved S1, radiologically evident screw loosening was found in 10 (28.8%) patients (Table 3). In this subgroup analysis, there were no differences in PI and LL (both *p* > 0.05) between the two groups of patients (with and without screw loosening). Although the differences did not reach significance, there was a trend toward higher delta LL (postoperation minus that of preoperation) in patients with screws loosening and S1 involvement. (9.3 ± 12.8 versus 0.4 ± 12.5, *p* = 0.07). Moreover, in patients who gained LL through the DDS surgery, the rate of screw loosening was significantly lower than in those who had decreased LL (12.1% versus 46.1%, *p* = 0.03) (Table 4).

### 3.4. Further Management

Among the 87 loosened screws found in 42 patients, there were 4 screws broken (0.4%), which were found in 4 patients (1.9%). In the serial follow-up of these patients, there were few clinically significant symptoms. One patient who had a loosened screw later received secondary revision surgery due to progressive spondylolisthesis at the adjacent level, which was treated with transforaminal lumbar interbody fusion.

In the current series, there were no malpositioned screws that caused neurological symptoms which required revision surgery, although postoperative CTs demonstrated several breaches of the pedicles. There were 2 patients who had postoperative infection and received secondary surgery for the removal of implants within 2 years after the primary surgery.

## 4. Discussion

This study analyzed 1064 Dynesys screws used for dynamic stabilization in 206 patients with lumbar spondylosis. During a mean follow-up period of 51 months, 87 screws (8.2%) in 42 patients (20.4%) became loose. Interestingly, the clinical outcomes equally improved whether screw loosening was evident or not. Most of the patients with screw loosening were asymptomatic, and screw loosening was identified only radiographically during regular follow-ups, except one patient who actually received secondary surgery of fusion for spondylolisthesis at an adjacent level. The study demonstrated that quite a proportion of patients who received DDS actually had screw loosening, but this did not affect the clinical improvement over an average of 4 years. Furthermore, the present report indicated that older patients, construct involving S1, and those who had flat back (failed to gain LL) postoperatively were more likely to have screw loosening. This finding raised the concern of longer-term adverse events of this pedicle-based dynamic stabilization system. To date, this was the largest series of DDS with emphasis on screw loosening, clinical outcomes, and spinal pelvic alignment. The results could be reasonably anticipated because of the tendency of osteopenia in the elderly, structural characteristics of the S1 vertebra, and the importance of restoration of LL in lumbar spine surgery.

The problem of screw loosening is a concern in the dynamic stabilization system. Wu et al. [15] studied retrospectively 658 screws in 126 patients who underwent DDS for a mean follow-up of 37.0 months: 4.7% of screws in 19.8% patients had screw loosening. Ko et al. [14] studied retrospectively 71 patients who underwent decompression using DDS for 1- or 2-level lumbar spondylosis. Screw loosening in 19.7% of patients and 4.6% of screws was noted. In a series by Payer et al. [2] of 30 patients who had single-level degenerative lumbar disease with stenosis and who underwent DDS, screw loosening was found in 2 cases. Segura-Trepichio et al. [12] also reported on 22 patients who underwent DDS. A total of 4 (18%) patients had signs of loose screws. The rate of screw loosening in our report seems to be similar with previous studies.

In a previous report by Wu et al., patients of older age or those with diabetes were noted to have higher rates of screw loosening [15]. A large series published previously described decreased bone mineral density (BMD) associated with age [18, 19]. Decreased bone mineral density, especially in patients with osteoporosis, is indicative of a high risk of screw loosening. On the other hand, there was no statistically significant change of screw loosening rates in patients with diabetes mellitus (DM) between the groups in our present study. For patients with type 2 DM, some authors have reported an elevated BMD [20–22], while other studies have reported a decreased BMD [23, 24], and some have reported unaltered bone density [20, 25]; some cross-sectional studies have even found normal BMD [26, 27]. The effect of DM on BMD or screw loosening is controversial.

From a biomechanical viewpoint, the lumbosacral junction has a high mechanical demand and short, wide, cancellous pedicles at L5 and S1 [28]. The lumbosacral junction and the disc level of L4/5 contribute the most to the formation of lumbar lordosis when compared to other disc levels. Bicortical fixation with S1 screws has been recommended to achieve adequate fixation at the lumbosacral junction [29]. The bridged segments of the lumbosacral junction is a close linkage system. In patients with lumbosacral fusion, distraction instrumentation would cease lumbar lordosis and cause flat back syndrome [30]. Kostuik and Hall [31] reviewed the cases of 45 adult patients in whom fusion was performed on the sacrum for scoliosis. Of those patients, 22 (49%) were noted to have lost lumbar lordosis. Thirteen (29%) underwent corrective osteotomies, with improvement in their pain. In our present study, all patients had clinical improvement in their VAS scores of the back and leg. Loss of lumbar lordosis with instrumentation presented no sign of flat back syndrome, but a higher screw loosening rate. We supposed that loss of lumbar lordosis in dynamic stabilization would cause a higher rate of screw loosening.

Pelvic incidence (PI), a spinal pelvic parameter, plays an important role in spinal sagittal balance. It is considered as a constant value decided by the individual anatomical position of the pelvis [32]. This parameter strongly correlates with lumbar lordosis by statistical analysis. The lower value of PI implies a flattened lordosis. For example, Boulay et al. in 2006 conducted a study about the correlation between PI and lordosis [33]. The mean PI in adults is measured as 53 ± 9 degrees. A low PI value is considered as less than 44 degrees, which would lead to a flattened lordosis. A high PI (more than 62 degrees) would lead to more pronounced lordosis. Theoretically, a flattened lordosis contributes to a worse sagittal balance of the spine, and the standing position of an individual with inadequate LL would not be in the condition of efficient biomechanical economy.

In the current study, the screw loosening did not directly correlate with PI or lordosis (*p* > 0.05). However, it was correlated with the difference between pre- and postoperative lordosis. If the lumbar lordosis is flattened postoperatively, the screw loosening rate will increase significantly. The ideal positive or negative difference between PI and lordosis should be within 11 degrees [34]. The postoperative flattened lordosis would lead to a discordant balance to PI. Though this finding does not have clinical impact, the biomechanical effect cannot be ignored in the long run.

There are limitations to the current study. First, it is a retrospective study and inevitably had some loss during follow-up. However, all the patients in the series were treated by the same group of surgeons under a uniform management strategy. Thus, there was little selection bias between the patients involved in the current study. With a mean follow-up of more than 4 years (51 months), over one thousand Dynesys screws were followed up in more than 70% of the patients (>200 patients). This was the largest series to date specifically focused on Dynesys screw loosening. An additional analysis included all 291 patients of the current series and demonstrated that overall 21.3% of patients had screw loosening at a mean follow-up of 32.6 months. This comprehensive rate of screw loosening was similar to that analyzed from those patients who had more than 24 months of follow-up (i.e., 20.4% of patients at 40.7 months). The overall rate of loosening per screw, 8.4% at 32.6 months, was similar to that of 206 patients who had follow-up more than two years (i.e., 8.2% of screws at 40.7 months) (Table 5). Therefore, the data were not skewed when only patients with more than two years of follow-up were analyzed. The report reasonably reflected the actual clinical scenario.

Second, the determination of screw loosening was not ideal. The current method of identification of loosened Dynesys screws was dependent on the observer, radiologists, and neurosurgeons interpreting the image studies and thus could be arbitrary. The true identification of screw loosening should involve surgical exploration and pathological examination for confirmation of the weakened bone-screw interface. Nevertheless, the current study incorporated CT scans in conjunction with the dynamic radiographs. Although this assessment of screw loosening was not perfect, it was the best currently available evidence and also clinically practical. There were no comparable studies of such a scale in the literature assessing DDS with CTs and radiographs. Furthermore, the spinal pelvic parameters were seldom addressed in previous reports of DDS. It was reasonable to infer that PI and LL were critically important in motion preservation surgery of the lumbar spine. Also, this study did not demonstrate the actual timing of occurrence of the screw loosening, which could happen earlier than that caught on imaging studies due to clinical silence. These issues would require future studies with longer follow-up to clarify.

## 5. Conclusion

In patients who underwent DDS, the screw loosening rate was overall 8.2% of screws and 20.4% of patients, at a mean follow-up of more than 4 years. Older patients, S1 involvement, and those who failed to gain LL postoperatively were at higher risk of screw loosening. Although the screw loosening was not symptomatic, this raised a concern about its long-term clinical effects.

## Figures and Tables

**Table 1:** Demographic and clinical characteristics.

Characteristic	Value
Number of patients	206
Sex	
Male	115 (55.8%)
Female	91 (44.2%)
Age (year)^*∗*^	61.0 ± 12.9
Months of follow-up^*∗*^	
Imaging	40.7 ± 19.7
Clinical	51.1 ± 20.8
Number of instrumented levels	
1 level	86 (41.7%)
L2-3	2
L3-4	7
L4-5	61
L5-S1	16
2 levels	120 (58.3%)
L2-3-4	4
L3-4-5	86
L4-5-S1	30

^*∗*^Values are presented by mean ± SD.

**Table 2:** Comparison of patients with and without screw loosening.

Characteristic	Screw loosening		*p* value
	Yes	No	
Number of patients	42 (20.4%)	164 (79.6%)	
Age (year)^*∗*^	64.6 ± 11.6	60.1 ± 13.1	**0.03**
Sex (F : M)			0.99
Male	23	92	
Female	19	72	
BMI^*∗*^	24.6 ± 3.2	25.6 ± 3.8	0.22
DM	11/42	30/164	0.47
HTN	16/42	75/164	0.47
Smoke	3/42	15/164	0.92
Instrumentation			0.48
1 level	15/42	71/164	
2 levels	27/42	135/164	
Blood loss (mL)^*∗*^	822.6 ± 521.6	703.6 ± 544.5	0.22
Number of screws	87 (8.2%)	977 (91.8%)	
Screw distribution			
L2	0 (0%)	12 (100%)	
L3	16 (8.1%)	182 (91.9%)	
L4	24 (6.4%)	352 (93.6%)	
L5	32 (8.3%)	354 (91.7%)	
S1	15 (16.3%)	77 (83.7%)	

^*∗*^Values are presented by mean ± SD.

BMI = body mass index; DM = diabetes mellitus; and HTN = hypertension.

**Table 3:** Comparison of radiographic measurements in patients with S1 involvement.

Characteristic	Screw loosening		*p* value
	Yes	No	
Number of patients	10 (28.8%)	36 (71.2%)	
PI^*∗*^	40.8 ± 12.1	41.6 ± 9.9	0.84
LL^*∗*^			
Pre-op	29.9 ± 12.2	26.6 ± 9.9	0.45
Post-op	30.3 ± 11.9	35.9 ± 11.3	0.20
Delta (post-op minus pre-op)	0.4 ± 12.5	9.3 ± 12.8	0.07
PI − LL	10.9 ± 13.2	15.0 ± 12.1	0.39

^*∗*^Values are presented by mean ± SD.

PI = pelvic incidence; LL = lumbar lordosis.

**Table 4:** Rate of screw loosening in Dynesys dynamic stabilization involving S1.

	Screw loosening (Number of patients)		Rate of loosening	*p* value
	Yes	No		
Delta LL ≥0	4	29	12.1%	**0.03**
Delta LL <0	6	7	46.1%	

Total	10	36		

Delta LL (lumbar lordosis) was defined as postoperative LL minus preoperative LL.

**Table 5:** The comparison outcome of total patients and patients over 2-year follow-up.

	Number of patients	Follow-up (months)		Rate of loosening per patient (%)	Rate of loosening per screw (%)
		Clinical	Radiological		
All patients	291	46.4 ± 22.7	32.6 ± 21.2	21.3	8.4
Follow-up >2 years	206	51.1 ± 20.8	40.7 ± 19.7	20.4	8.2

## Abbreviations

ASD: Adjacent segment disease
BMD: Bone mineral density
BMI: Body mass index
CT: Computed tomography
DDD: Degenerative disc disease
DDS: Dynesys dynamic stabilization
DM: Diabetes mellitus
HTN: Hypertension
JOA: The Japanese Orthopaedic Association (JOA) scores
LL: Lumbar lordosis
MRI: Magnetic resonance image
ODI: Oswestry Disability Index
PI: Pelvic incidence
SMPS: Spinal motion preservation surgery
VAS: Visual Analogue Scale.
